# Orchestration of lincRNA-p21 and miR-155 in Modulating the Adaptive Dynamics of HIF-1α

**DOI:** 10.3389/fgene.2020.00871

**Published:** 2020-08-18

**Authors:** Cheng-Yuan Sun, Xiao-Peng Zhang, Feng Liu, Wei Wang

**Affiliations:** ^1^National Laboratory of Solid State Microstructures, Department of Physics, Nanjing University, Nanjing, China; ^2^Kuang Yaming Honors School, Nanjing University, Nanjing, China; ^3^Institute for Brain Sciences, Nanjing University, Nanjing, China

**Keywords:** hypoxia, HIF-1α, lincRNA-p21, miR-155, adaptive dynamics, feedback loop

## Abstract

Hypoxia-inducible factor-1 (HIF-1) is the key regulator of cellular adaptive response to hypoxia. Accumulating evidence shows that HIF-1 induces some non-coding RNAs (ncRNAs) including lncRNAs and miRNAs to modulate its own activity, enclosing several feedback loops. How the two classes of ncRNAs are orchestrated in the HIF-1-dependent adaptive response to hypoxia is poorly understood. By selecting lincRNA-p21 and miR-155 as the representatives, we develop an integrated model of the HIF-1 network comprising interlinked positive and negative feedback loops to clarify the interplay between the two ncRNAs in the hypoxic response. By numerical simulations, we find that coordination of lincRNA-p21 and miR-155 shapes the adaptive dynamics of HIF-1α: lincRNA-p21 induction in the early phase stimulates the upregulation of HIF-1α via stabilizing it, while miR-155 induction in the late phase promotes the recovery of HIF-1α via enhancing the degradation of its mRNA. Moreover, HIF-1α-induced PHD2 plays an auxiliary role in the decline of HIF-1α. In addition, lincRNA-p21 and miR-155 modulate each other via regulating HIF-1α activity. Together, lincRNA-p21 and miR-155 coordinate in modulating HIF-1α dynamics, and our work may shed light on the role for ncRNAs in the cellular adaptation to hypoxia.

## 1. Introduction

Hypoxia plays significant roles in human physiology and diseases including cancer (Koh and Powis, 2012). Hypoxia-inducible factor-1 (HIF-1) is the key mediator of the cellular adaption to hypoxia (Schofield and Ratcliffe, 2004). HIF-1 is a heterodimer composed of an oxygen-dependent α-subunit (HIF-1α) and a constitutively expressed nuclear β-subunit (HIF-1β) (Wang et al., 1995). Under normoxia, HIF-1α is hydroxylated by prolyl hydroxylases (PHDs) on Pro402 and Pro564, and these modifications facilitate the binding of HIF-1α to VHL (von Hippel-Lindau), promoting the ubiquitin-dependent proteasomal degration of HIF-1α (Ohh et al., 2000; Jaakkola et al., 2001). In addition, the hydroxylase factor inhibiting HIF-1 (FIH-1) hydroxylates HIF-1α on Asn803 to repress its transcriptional activity via preventing the recruitment of coactivator p300/CBP (Mahon et al., 2001). Upon hypoxia, PHDs and FIH-1 are deactivated so that HIF-1α is stabilized and translocates to the nucleus to form a transcriptional complex with HIF-1β (Maxwell et al., 2001). Activated HIF-1 induces hundreds of genes involved in glycolysis, angiogenesis, cell survival, and metastasis (Harada et al., 2007; Semenza, 2009, 2012; Zeng et al., 2015). Moreover, HIF-1α itself shows adaptive dynamics in the hypoxic response (Stiehl et al., 2006; Minamishima et al., 2009). It has been reported that tight control of transient HIF-1α dynamics is essential for cell survival in hypoxia (Ginouvés et al., 2008; Henze et al., 2010; Bagnall et al., 2014). The detailed mechanism underlying the adaptive dynamics of HIF-1α in hypoxia is unclear.

MicroRNAs (miRNAs), especially HIF-1-inducible miRNAs, also play significant roles in cellular response to hypoxia (Serocki et al., 2018). For example, HIF-1-targeted miR-210 is shown to regulate cellular metabolism or angiogenesis during hypoxia (Chan et al., 2009; Li et al., 2016). miR-155 is induced by HIF-1 in multiple cell lines (Xie et al., 2015). It was found that miR-155 contributes to the descending of HIF-1α in the late phase by enhancing the degradation of HIF-1α mRNA, enclosing a negative feedback loop (Bruning et al., 2011). Moreover, HIF-1 upregulates the expression of PHD2 or PHD3 to promote HIF-1α degradation, compensating for repression of PHD activity in hypoxia (Minamishima et al., 2009; Bagnall et al., 2014). An intriguing question is whether miR-155 and PHDs play distinct roles in the downregulation of HIF-1α.

Long non-coding RNAs (lncRNAs) are also involved in the hypoxic response by regulating HIF-1 activity (Chang et al., 2016). LincRNA-p21 represses the degradation of HIF-1α by blocking the VHL-HIF-1α interaction, enclosing a positive feedback loop (Yang et al., 2014). As a result, HIF-1α amplifies its own activation and induces Glut1 and LDHA to facilitate glycolysis in hypoxic cells (Yang et al., 2014). Thus, there exist several HIF-1α-centered negative and positive feedback loops involving PHDs, miR-155, and lincRNA-p21. It is a challenge to clarify how these interlinked feedback loops interplay in shaping HIF-1α dynamics under distinct hypoxic conditions.

A series of theoretical models have been developed to explore the mechanism for the regulation of HIF-1α dynamics (Kohn et al., 2004; Qutub and Popel, 2006; Dayan et al., 2009; Nguyen et al., 2013). Kohn et al. explored the mechanism for the switch-like response of HIF-1 to hypoxia (Kohn et al., 2004). Qutub et al. characterized the effects of micro-environmental factors, such as ascorbate, iron, and PHD, on the hydroxylation of HIF-1α (Qutub and Popel, 2006). Nguyen et al. clarified the regulation of HIF-1α stability and activity by FIH-1 (Nguyen et al., 2013). We have explored the interplay between HIF-1α and p53 upon hypoxia in several models (Zhou et al., 2015; Wang et al., 2019; Ye et al., 2019). Although miRNAs-mediated HIF-1α regulation has been involved in some modeling studies (Bruning et al., 2011; Fábián et al., 2016), how HIF-1-targeted miRNAs and lncRNAs are orchestrated to regulate HIF-1α is less understood. It is feasible to select miR-155 and lincRNA-p21 as the representatives since they are both expressed at least in HeLa cells (Bruning et al., 2011; Yang et al., 2014). It is promising to clarify how the crosstalk between miR-155 and lincRNA-p21 modulates HIF-1α dynamics upon hypoxia by modeling.

Here, we develop a model of the HIF-1 signaling network including lincRNA-p21 and miR-155 to explore how different ncRNAs coordinate to mediate the adaptive response of HIF-1α to hypoxia. Our results show that lincRNA-p21 and miR-155 are induced in different phases of the response to shape the adaptative dynamics of HIF-1α. LincRNA-p21 induction in the early phase stabilizes HIF-1α by blocking its degradation, whereas miR-155 is induced in the late phase to downregulate HIF-1α via enhancing HIF-1α mRNA degradation. Moreover, miR-155 and PHD2 cooperate to facilitate the recovery of HIF-1α. We found that lincRNA-p21 and miR-155 compete with each other to modulate HIF-1α dynamics. Together, HIF-1 sequentially induces lincRNA-p21 and miR-155 to facilitate the cellular adaption to hypoxia.

## 2. Models and Methods

### 2.1. Overview of the Model

**Figure 1:**
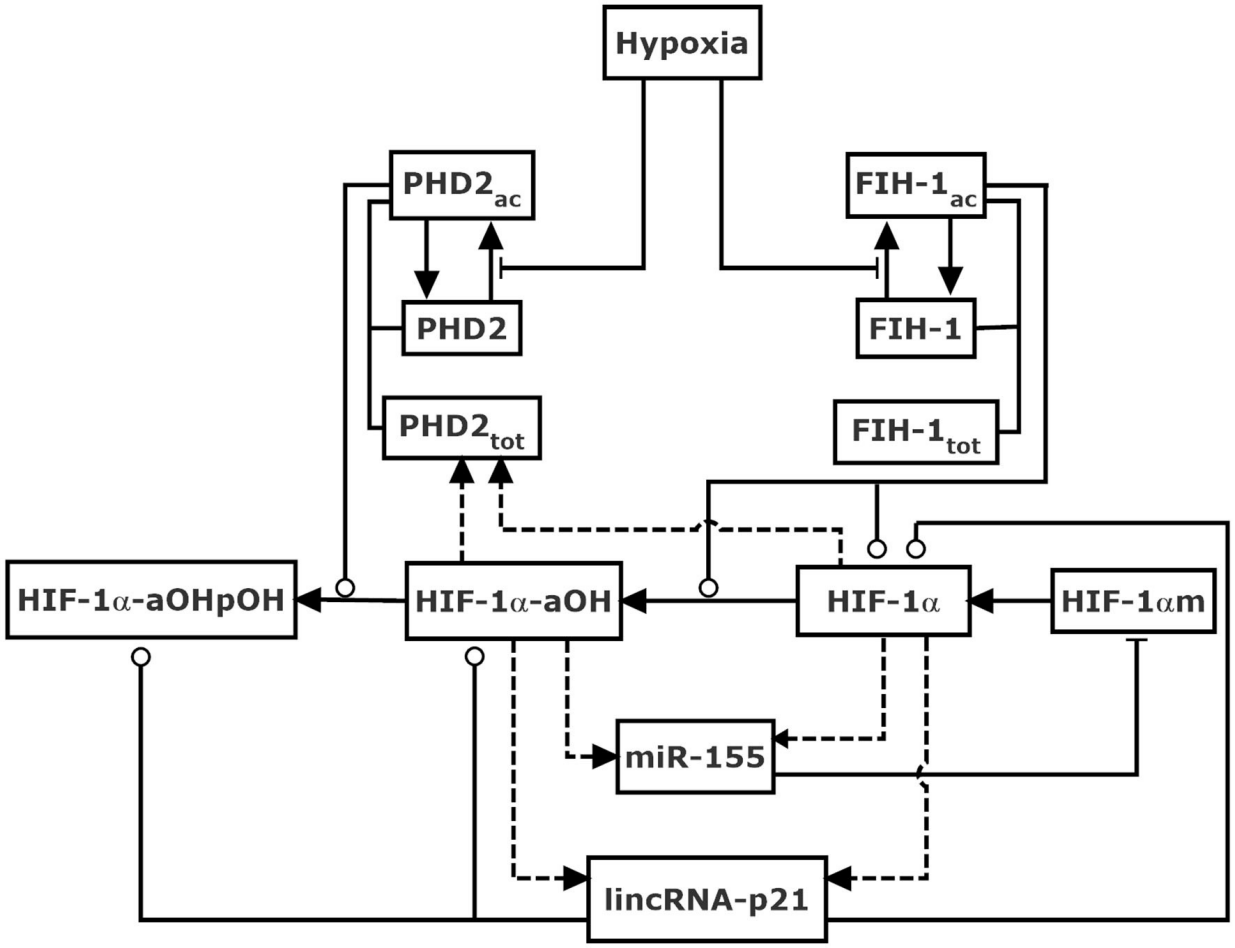
Schematic diagram of the model for HIF-1α network in response to hypoxia mediated by non-coding RNAs. HIF-1α is hydroxylated by PHD2 and FIH-1 upon hypoxia. HIF-1α-aOH and HIF-1α separately represent partially and fully activated form of HIF-1α, and both promote the induction of lincRNA-p21, miR-155, and PHD2. LincRNA-p21 enhances the stabilization of HIF-1α, while miR-155 promotes the degradation of HIF-1α mRNA, thereby enclosing interlocked positive and negative feedback loops, respectively. FIH-1 also modulates the degradation of HIF-1α. Dashed lines denote gene expression. Solid arrow-headed lines represent transitions between proteins. Circle-headed and bar-headed lines denote promotion and inhibition of enzymatic reactions, respectively.

We built an integrated model of the HIF-1 network in response to hypoxia, focusing on the role of lincRNA-p21 and miR-155 in shaping HIF-1 dynamics (Figure 1). For simplicity, subcellular compartmentalization is not considered. Given the constitutive expression of HIF-1β in the hypoxic response (Wang et al., 1995), the dimerization of HIF-1α and HIF-1β is ignored, and HIF-1 heterodimer is not distinguished from HIF-1α thereafter. The model is mainly composed of two modules responsible for oxygen sensing and feedback regulation of HIF-1α.

The stability and activity of HIF-1α is controlled by the sensors of oxygen, PHDs, and FIH-1, respectively. In our model, PHD2 is considered the representative of PHDs as it is the primary oxygen sensor among the three PHD isoforms (Berra et al., 2003; Takeda et al., 2006). We consider two forms of PHD2: PHD2 (inactive form) and PHD2_ac_ (active form); FIH-1 is divided into FIH-1 (inactive form) and FIH-1_ac_ (active form). Upon hypoxia, PHD2 and FIH-1 are deactivated, leading to the stabilization and activation of HIF-1α (Jaakkola et al., 2001; Mahon et al., 2001). It is assumed that the total amount of PHD-2, PHD2_tot_, is HIF-1α-dependent (Stiehl et al., 2006), whereas that of FIH-1, FIH-1_tot_, is supposed to be a constant since its expression is independent of HIF-1α.

HIF-1α protein is produced by the translation of HIF-1α mRNA (HIF-1αm). Three forms of HIF-1α protein are considered, i.e., HIF-1α (unhydroxylated), HIF-1α-aOH (asparagine-hydroxylated), and HIF-1α-aOHpOH (hydroxylated at both proline and asparagine sites). In other words, HIF-1α-aOHpOH is proline-hydroxylated form while HIF-1α and HIF-1α-aOH are proline-unhydroxylated forms. Of note, the hydroxylation steps are supposed to be irreversible (Schofield and Ratcliffe, 2004; Chan et al., 2005). Although the hydroxylation of HIF-1α by FIH-1 represses its transcriptional activity via preventing the recruitment of co-activator p300 (Lando et al., 2002), we assume that HIF-1α with asparagine-hydroxylation alone is of partial transcriptional activity (Dayan et al., 2006; Chan et al., 2016). Thus, it is assumed both HIF-1α and HIF-1α-aOH can induce miR-155, lincRNA-p21, and PHD2. miR-155 regulates HIF-1α posttranscriptionally by promoting the degradation of HIF-1α mRNA (Bruning et al., 2011), while lincRNA-p21 can promote the stabilization of HIF-1α (Yang et al., 2014). Together, two negative and one positive feedback loops are interlinked to regulate HIF-1α.

### 2.2. Details of the Model

The network model is described by a set of ordinary differential equations. The key points for the equations are listed as follows. The production rate of HIF-1α mRNA is assumed to be a constant, while its degradation rate is described by Michaelis-Menten dynamics depending on miR-155 level (Equations 1–2). The oxygen-dependent activation of PHD2 and FIH-1 is described by Michaelis-Menten kinetics (Equations 3, 4, 9, and 10). In addition, the disassociation constant of FIH-1 for oxygen is markedly lower than that of PHD2 for oxygen (Koivunen et al., 2004), thus the threshold level of oxygen in FIH-1 activation is set to be much smaller than that in PHD2 activation (see Table S2). The hydroxylation of HIF-1α by PHD2_ac_ and FIH-1_ac_ is also depicted by Michaelis-Menten kinetics (Equations 5–7).

Given the disassociation constant of FIH-1 for oxygen is much lower than that of PHD2, HIF-1α should be preferentially asparagine-hydroxylated by FIH-1 (Koivunen et al., 2004). It has been identified that proline-hydroxylation promotes the oxygen-dependent degradation of HIF-1α (Ivan et al., 2001). For the above two reasons, HIF-1α hydroxylated at proline residues alone is omitted. In addition, we assumed that there exists a PHD2-independent degradation of unhydrxoylated HIF-1α that is repressed by FIH-1_ac_ since asparaginyl hydroxylation may protect HIF-1α from oxygen-independent degradation (Nguyen et al., 2013) (Equation 5).

The induction rates of miR-155, lincRNA-p21, and PHD2 by HIF-1α and HIF-1α-aOH are all characterized by Hill functions (Equations 8, 11, and 12). Moreover, lincRNA-p21 can further the stabilization of HIF-1α by blocking its interaction with VHL that acts as a ubiquitin E3 ligase for HIF-1α degradation (Yang et al., 2014). For simplicity, the processes of the VHL-HIF-1α interaction and HIF-1α ubiquitination are not explicitly considered, and the degradation rate of HIF-1α is depicted by Michaelis-Menten kinetics depending on lincRNA-p21 levels (Equations 5–7). We assume that the rate constant for the degradation of proline-unhydroxylated HIF-1α is much lower than that for proline-hydroxylated HIF-1α (i.e., *k*_dhif_ ≪ *k*_dhifpoh_) since VHL mainly interacts with proline-hydroxylated HIF-1α for oxygen-dependent degradation (Ivan et al., 2001) (Equations 5–7). Nevertheless, we still consider the effect of lincRNA-p21 on the stabilization of proline-unhydroxylated HIF-1α via blocking the binding of VHL. Moreover, the effect of lincRNA-p21 on the interaction between VHL and HIF-1α may be not significantly affected by hydroxylation (Yang et al., 2014). Thus, the Michaelis constants of lincRNA-p21 for repressing the degradation of proline-hydroxylated HIF-1α (*j*_dhifpoh_) and proline-unhydroxylated HIF-1α (*j*_dhif_) are assumed to be equal.

### 2.3. Methods

The concentration of each species is represented by [.], corresponding to a state variable in rate equations in Supporting Material. The relative value of the oxygen level is adopted, and 1 represents 1% *O*_2_ in the model. The reactions concerned with hydroxylation or activation are described by Michaelis-Menten kinetics. The depiction of variables and their initial values are listed in Table S1. All the initial values of the variables are set to be their steady states under normoxia. The standard values of the parameters are listed in Table S2. The unit of time is minutes and the units of parameters are decided so that the concentration of proteins or RNAs is dimensionless. The ordinary differential equations were solved numerically by Oscill8 (http://oscill8.sourceforge.net/) with adaptive time steps. The bifurcation diagrams were also plotted using Oscill8.

## 3. Results

### 3.1. Overview of HIF-1α Dynamics Upon Hypoxia

**Figure 2:**
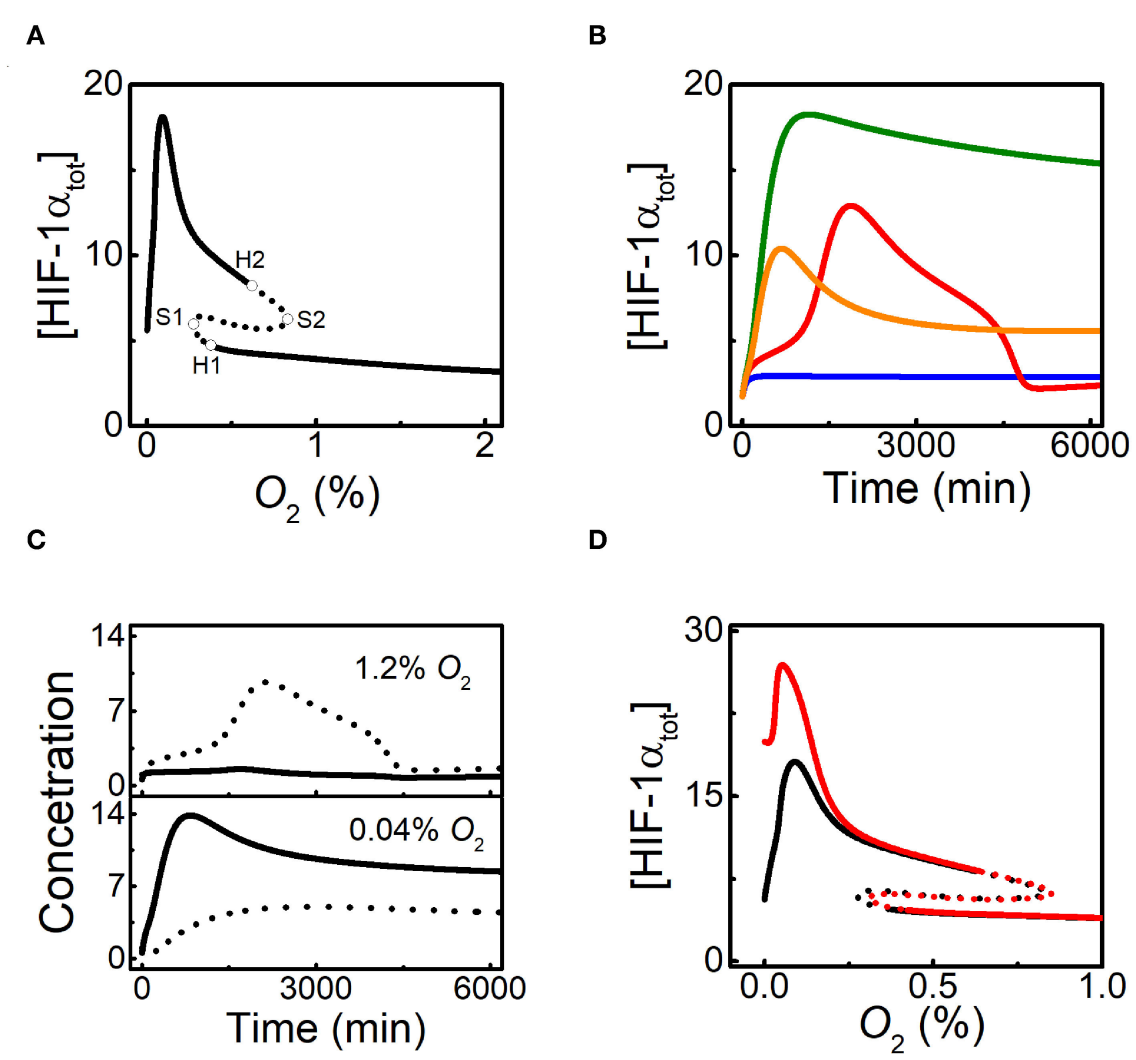
HIF-1α dynamics under different hypoxic conditions. **(A)** Bifurcation diagrams of [HIF-1α_tot_] vs. *O*_2_%, in the default parameters setting. The stable and unstable steady states are indicated by solid and dashed lines, respectively. The saddle-node bifurcation points are marked with S1 and S2, while the Hopf bifurcation points are labeled with H1 and H2. **(B)** Time courses of [HIF-1α_tot_] for 3% (blue), 1% (red), 0.05% (green), and 0% *O*_2_ (orange). The initial values of all the species are set to be their steady states at 21% *O*_2_ in the simulation of the dynamics of the species (the same below). **(C)** Time courses of [HIF-1α] (solid) and [HIF-1α-aOH] (dashed) in moderate hypoxia (1.2% *O*_2_) or severe hypoxia (0.04% *O*_2_). **(D)** Bifurcation diagrams of [HIF-1α_tot_] vs. *O*_2_ level for *k*_dhiffih_=0.0032 (black) or 0 (red). Notably, *k*_dhiffih_ is designated as FIH-1-related degration rate of HIF-1α. The types of bifurcation points are similar to Figure 2B.

We first display the dependence of the total level of HIF-1α, [HIF-1α_tot_], on *O*_2_ level by bifurcation diagram (Figure 2A). The response curve is divided into several parts by four bifurcation points including two saddle-node bifurcation points (S1 and S2) and two Hopf bifurcation points (H1 and H2). The two branches separated by S1 and S2 correspond to the low and high states of [HIF-1α_tot_]. With decreasing *O*_2_ levels, the stable level of HIF-1α_tot_ increases slowly. The steady states of [HIF-1α_tot_] become unstable for *O*_2_ levels between H1 and S1. [HIF-1α_tot_] switches to high stable states in this regime and its stable- state level rises continuously until it reaches the maxima around 0.1% *O*_2_, then drops to low levels under severe hypoxia, which is qualitatively in accordance with the experimental data (Jiang et al., 1996). In addition, the high steady states of [HIF-1α_tot_] exhibit instability for *O*_2_ levels between H2 and S2.

Under different *O*_2_ levels, the temporal dynamics of [HIF-1α_tot_] are shown in Figure 2B. For mild hypoxia (3% *O*_2_), [HIF-1α_tot_] keeps at low levels; for moderate hypoxia (1% *O*_2_), [HIF-1α_tot_] exhibits adaptive dynamics, which is related to the existence of Hopf bifurcation points; for severe hypoxia (0.05% *O*_2_), [HIF-1α_tot_] eventually reaches a high level; for anoxia (0% *O*_2_), [HIF-1α_tot_] shows a smaller pulse and drops to lower levels finally. Of note, when O_2_ level is between H1 and S1 (0.3% *O*_2_), [HIF-1α_tot_] first climbs to a very high level and then settles down to a fairly high level (Figure S1), consistent with the instability of low steady states (see Figure 2A).

As mentioned above, FIH-1 preferentially hydroxylates HIF-1α and can maintain its activity at lower oxygen levels than PHD2 (Koivunen et al., 2004). As a result, PHD2 and FIH-1 are deactivated sequentially under aggravating hypoxia. Our results show that [HIF-1α-aOH] and [HIF-1α] are predominant under moderate and severe hypoxia, respectively (Figure 2C). For moderate hypoxia, [HIF-1α-aOH] exhibits pulsatile dynamics and is much higher than [HIF-1α], while fully activated HIF-1α becomes dominant under severe hypoxia. Therefore, HIF-1α is progressively activated in response to hypoxia.

As shown in Figure 2A, [HIF-1α] drops markedly under anoxia, consistent with experimental results (Jiang et al., 1996). Figure 2D shows the bifurcation diagrams of [HIF-1α_tot_] vs. *O*_2_% with or without FIH-1-mediated degradation. The two diagrams are separable only in severe hypoxia and anoxia, which means that FIH-1 protects HIF-1α from degradation only under such conditions. HIF-1α accumulates markedly and its level decreases mildly in the absence of FIH-1-mediated degradation. The marked decline of [HIF-1α] should result from FIH-1 deactivation that facilitates HIF-1α degradation under severe hypoxia or anoxia. Our results may provide a plausible mechanism for the regulation of HIF-1α degradation independent of PHDs.

### 3.2. HIF-1α-Induced lincRNA-p21 Modulates the Adaptive Dynamics of HIF-1α Through a Positive Feedback Loop

**Figure 3:**
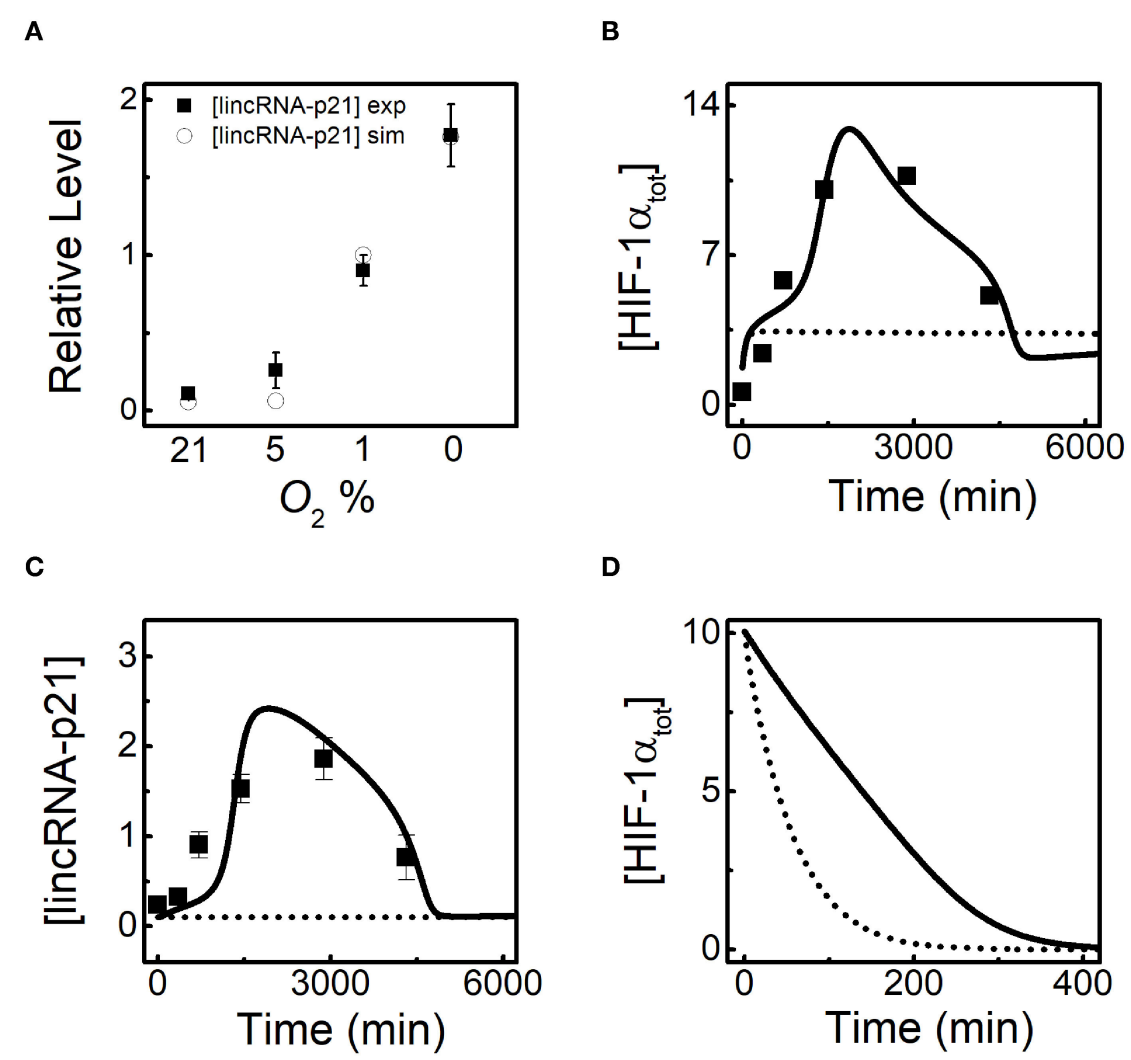
LincRNA-p21 is required for the adaptive dynamics of HIF-1α. **(A)** Comparison of [lincRNA-p21] at 24 h for different *O*_2_ levels between simulation (circle) and experiment (black square). The experimental data are retrieved from Yang et al. (2014). **(B,C)** Time courses of [HIF-1α_tot_] **(B)** and [lincRNA-p21] **(C)** with normal lincRNA-p21 expression (solid, *k*_sLAp21_ = 0.08 and *k*_sLAp21a_ = 0.05) and knockout (dashed, *k*_sLAp21_ = 0 and *k*_sLAp21a_ = 0) at 1% *O*_2_. Black squares denote the experimental data for [HIF-1α_tot_] and [lincRNA-p21] with normal lincRNA-p21 expression, retrieved from Yang et al. (2014). **(D)** With inhibited protein synthesis (*k*_thif_ = 0), time courses of [HIF-1α_tot_] with normal lincRNA-p21 expression (solid, *k*_sLAp21_ = 0.08 and *k*_sLAp21a_ = 0.05) and knockout (dashed, *k*_sLAp21_ = 0 and *k*_sLAp21a_ = 0) under 1% *O*_2_. Of note, the initial state refers to the transient state at 24 h under 1% *O*_2_ in the standard parameter setting.

HIF-1α induces lincRNA-p21 to promote its own stabilization (Yang et al., 2014), and how the latter modulates the adaptive dynamics of HIF-1α is investigated in the following. To further verify our model, we compare the simulation results for [lincRNA-p21] at 24h with the experimental results for different *O*_2_ levels (Yang et al., 2014). LincRNA-p21 is indeed markedly evoked in hypoxia, showing good agreements with the experimental data (Figure 3A). Both [HIF-1α_tot_] and [lincRNA-p21] exhibit adaptive dynamics at 1% O_2_ in the standard parameter setting, and the results are well consistent with experimental data (Yang et al., 2014) (Figures 3B,C). When HIF-1α-dependent lincRNA-p21 expression is removed, [HIF-1_tot_] remains at rather low levels, meaning that lincRNA-p21 induction is crucial for the accumulation of HIF-1α in the ascending phase. In addition, given translation inhibition and the high initial level of HIF-1α_tot_, lincRNA-p21 knockout makes [HIF-1α_tot_] decay much faster compared with the normal case at 1% *O*_2_ (Figure 3D). These results show that lincRNA-p21 upregulates HIF-1α by repressing its degradation (Yang et al., 2014; Meng et al., 2018). Together, lincRNA-p21 is required for the adaptive dynamics of HIF-1α.

**Figure 4:**
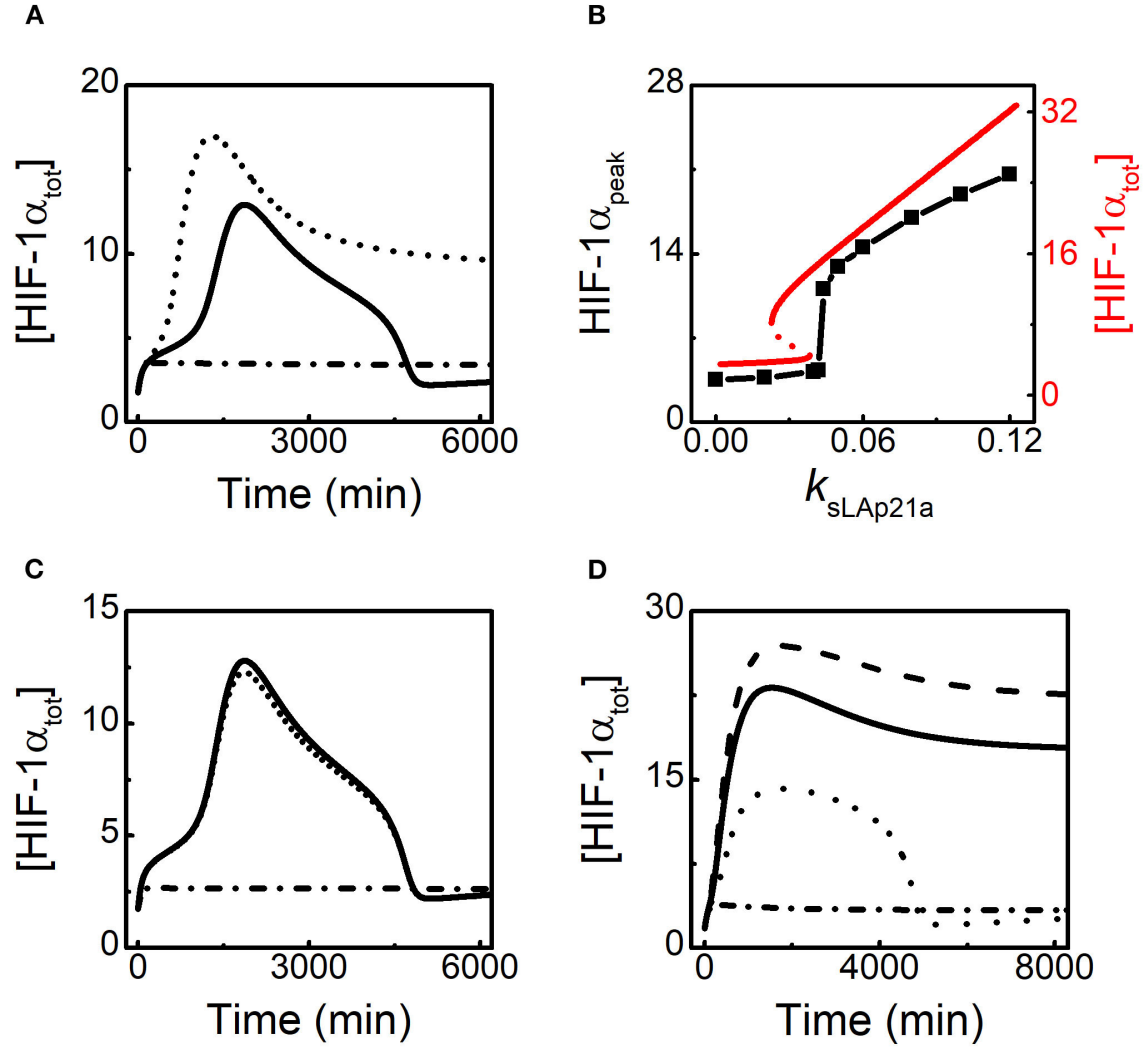
HIF-1α induces lincRNA-p21 to promote its own accumulation in hypoxia. **(A)** Time courses of [HIF-1α_tot_] at 1% *O*_2_ for different HIF-1α-aOH-dependent lincRNA-p21 induction rates: *k*_sLAp21a_ = 0.08 (dashed), 0.05 (solid, default), and 0 (dash-dotted). **(B)** The curve of HIF-1α_peak_ (black) vs. *k*_sLAp21a_ at 1% *O*_2_; the bifurcation diagram of [HIF-1_tot_] (red) vs. *k*_sLAp21a_ at 1% *O*_2_ with miR-155 knockout (*k*_smiR1551_ = 0 and *k*_smiR1551a_ = 0). HIF-1α_peak_ denotes the maximal of [HIF-1α_tot_] on individual simulation trials. **(C)** Time courses of [HIF-1α_tot_] at 1% *O*_2_ in the following cases: *j*_dhifpoh_ = 0.3 and *j*_dhif_ = 0.3 (solid); increasing *j*_dhifpoh_ to 100 (dashed); increasing *j*_dhif_ to 100 (dash-dotted). **(D)** Time courses of [HIF-1_tot_] at 0.1% *O*_2_ for different HIF-1α-dependent lincRNA-p21 synthesis rates: *k*_sLAp21_ = 0.12 (dashed), 0.08 (solid, default), 0.035 (dotted), and 0 (dash-dotted).

Given lincRNA-p21 is induced by HIF-1α, we further explore the effect of lincRNA-p21 induction rate on HIF-1α adaptation to hypoxia. Since HIF-1α-aOH is predominant under moderate hypoxia (Figure 2C), we only consider the effect of HIF-1α-aOH-dependent induction rate of lincRNA-p21 (*k*_sLAp21a_) on HIF-1α dynamics (Figures 4A,B). At 1% *O*_2_, as mentioned above, [HIF-1α_tot_] displays adaptive dynamics; for *k*_sLAp21a_ = 0, HIF-1α cannot be evoked; for rather large *k*_sLAp21a_ (0.08), although [HIF-1α_tot_] rises more sharply in the early phase, it settles at fairly high levels instead of dropping to low levels (Figure 4A). Therefore, proper expression of lincRNA-p21 is required for the prefect adaptation of HIF-1α to hypoxia.

The peak during the temporal evolution of [HIF-1α_tot_], HIF-1α_peak_, is selected as another indicator to show the effect of lincRNA-p21 abundance on HIF-1α dynamics. We find that the induction rate of lincRNA-p21, *k*_sLAp21a_, affects HIF-1 induction in a switch-like way (Figure 4B). For small *k*_sLAp21a_, HIF-1α_peak_ keeps rather small. But when *k*_sLAp21a_ is increased and exceeds some threshold, HIF-1α_peak_ rises sharply. With further increasing *k*_sLAp21a_, HIF-1α_peak_ rises continuously. Thus, the peak of [HIF-1α_tot_] is remarkably modulated by lincRNA-p21 abundance. To explain these results, we plot the bifurcation diagram of [HIF-1α_tot_] vs. *k*_sLAp21a_ with miR-155 knockout at 1% *O*_2_ (Figure 4B). Due to the HIF-1α-lincRNA-p21 positive feedback loop, the steady state of [HIF-1α_tot_] exhibits bistability with varying *k*_sLAp21a_ and the threshold of *k*_sLAp21a_ is very close to that in the curve of HIF-1α_peak_. For *k*_sLAp21a_ exceeding the threshold, the steady state of [HIF-1α_tot_] is close to HIF-1α_peak_ and increases monotonically with increasing *k*_sLAp21a_. Therefore, HIF-1α is augmented by its target lincRNA-p21 in the rising phase in response to hypoxia.

We further analyze how lincRNA-p21-mediated HIF-1α stabilization affects its dynamics. The thresholds of lincRNA-p21 for repressing the degradation of proline-hydroxylated and -unhydroxylated HIF-1α are represented by *j*_dhifpoh_ and *j*_dhif_, respectively. [HIF-1α_tot_] remains at rather low levels when *j*_dhif_ is enlarged markedly (Figure 4C). Inhibiting the stabilization of proline-unhydroxylated HIF-1α by lincRNA-p21 markedly influences [HIF-1α_tot_] since both HIF-1α and HIF-1α-aOH are destabilized remarkably in this case. In contrast, for very large *j*_dhifpoh_, [HIF-1α_tot_] still exhibits adaptive dynamics perfectly which is close to the case in the standard parameter setting. The proline-hydroxylated HIF-1α-aOHpOH is rather unstable due to inhibition of its stabilization via lincRNA-p21. Therefore, HIF-1α induces lincRNA-p21 to facilitate its own accumulation in the adaptive response to moderate hypoxia.

HIF-1α adapts to moderate hypoxia while it remains at rather high levels under severe hypoxia (see Figure 2B). The effect of lincRNA-p21 induction rate on HIF-1α dynamics is investigated. Since lincRNA-p21 is mainly induced by un-hydroxylated HIF-1α in this case, we only consider the influence of the production rate of lincRNA-p21, *k*_sLAp21_, on HIF-1α dynamics (Figure 4D). In the default case, HIF-1α stays at high levels after a slight decrease at 0.1%O_2_. For increased *k*_sLAp21_, HIF-1α_tot_ rises to higher levels. For smaller *k*_sLAp21_, [HIF-1α_tot_] can exhibit an adaptive pulse with a lower amplitude. However, for very small *k*_sLAp21_, HIF-1α is hardly induced. Together, lincRNA-p21 is also required for HIF-1 accumulation under severe hypoxia and its induction rate can modulate the dynamic modes of HIF-1α.

### 3.3. HIF-1α Induces miR-155 to Promote Its Own Recovery in the Late Phase

**Figure 5:**
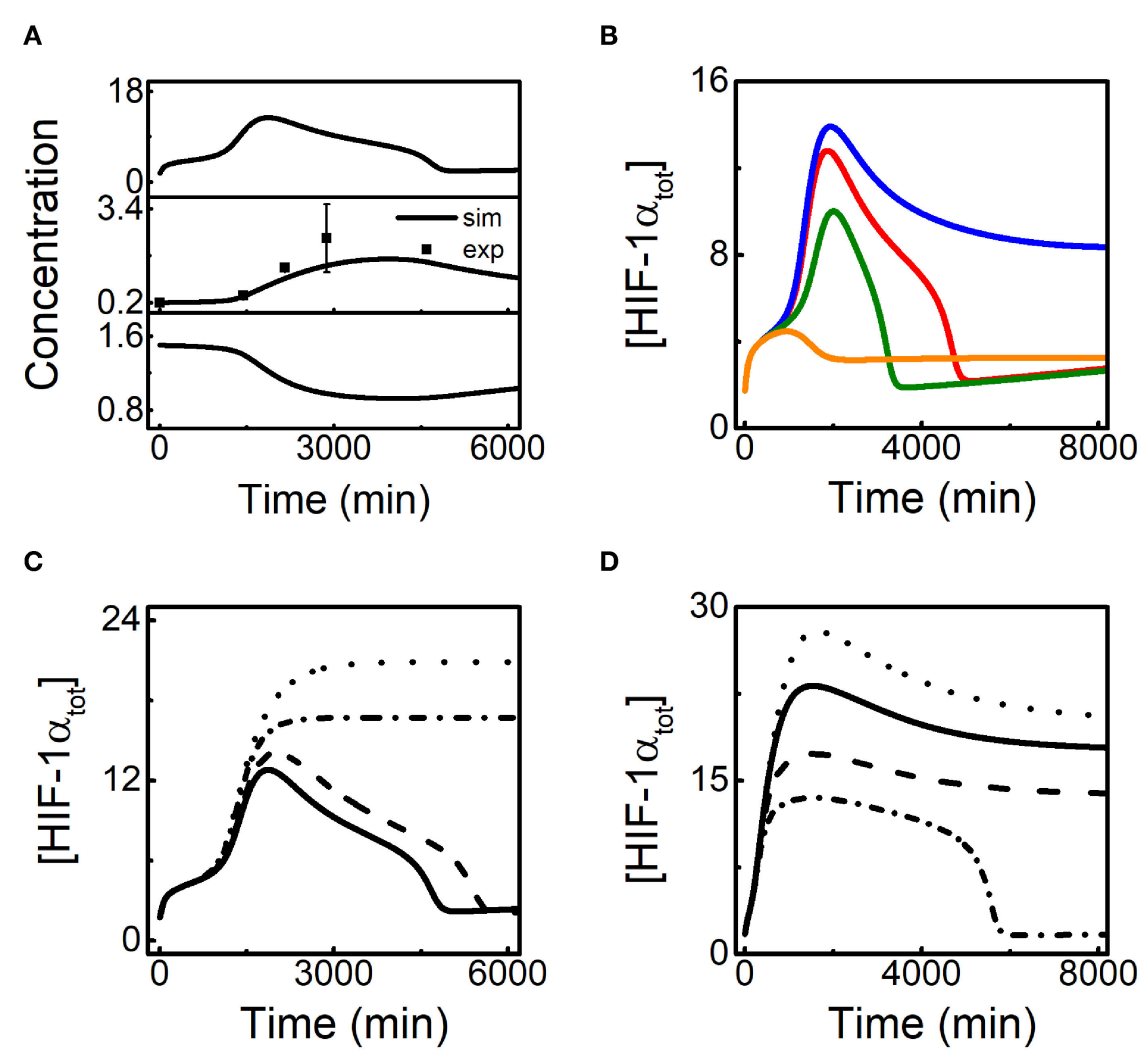
HIF-1α-induced miR-155 promotes the recovery of HIF-1α in the late phase of the adaptive response. **(A)** Time courses of [HIF-1α_tot_], [miR-155], and [HIF-1αm] from top to bottom at 1% *O*_2_, and the experimental data (retrieved from Bruning et al., 2011; Wan et al., 2014) are denoted by the black squares. **(B)** Time courses of [HIF-1α_tot_] at 1% *O*_2_ for different HIF-1α-aOH-dependent miR-155 induction rates: *k*_smiR1551a_ = 0.0006 (blue), 0.0012 (red, default), 0.0036 (green), and 0.007 (brown). **(C)** Time courses of [HIF-1α_tot_] at 1% *O*_2_ with miR-155 and PHD2 double knockout (dotted, *k*_smiR1551_ = 0, *k*_smiR1551a_ = 0, *k*_sphd1_ = 0, and *k*_sphd1a_ = 0), miR-155 knockout (dash-dotted, *k*_smiR1551_ = 0 and *k*_smiR1551a_ = 0), PHD2 knockout (dashed, *k*_sphd1_ = 0 and *k*_sphd1a_ = 0), and normal miR-155 and PHD2 expression. **(D)** Time courses of [HIF-1α_tot_] at 0.1% *O*_2_ for different HIF-1α-dependent miR-155 induction rates: *k*_smiR1551_ = 0.0006 (dotted), 0.0024 (solid, default), 0.012 (dashed), and 0.04 (dash-dotted).

It has been reported experimentally that HIF-1α-induced miR-155 can promote the recovery of HIF-1α in several cell lines (Bruning et al., 2011). The time courses of [HIF-1α_tot_], [miR-155], and [HIF-1αm] in hypoxia (1% *O*_2_) are shown in Figure 5A. HIF-1α_tot_ rises and induces miR-155, which promotes the degradation of HIF-1α mRNA so that both HIF-1α mRNA and HIF-1α_tot_ exhibit adaptive dynamics with some phase difference. As a result, miR-155 also drops to basal levels in the late phase, well consistent with the experimental data (Bruning et al., 2011; Wan et al., 2014). Next we explore the effect of HIF-1α-aOH-dependent induction rate of miR-155 (*k*_smiR1551a_) on the adaptive response of HIF-1α to hypoxia (Figure 5B). For decreased *k*_smiR1551a_, the adaptive property of [HIF-1α_tot_] dynamics weakens remarkably: HIF-1α_tot_ reaches a slightly higher peak and maintains at rather high levels finally. For increased *k*_smiR1551a_, [HIF-1α_tot_] still exhibits adaptive dynamics while the peak and the width of the dynamic curves reduce gradually and [HIF-1α_tot_] only rises slightly for very large *k*_smiR1551a_ (Figure 5B). Together, miR-155 can facilitate the recovery of HIF-1α levels in the late phase.

In addition to miR-155, HIF-1α-induced PHD2 may also contribute to the adaptation of HIF-1α to hypoxia (Stiehl et al., 2006; Henze et al., 2010; Bagnall et al., 2014). We further explore the potential interplay between miR-155 and PHD2 in regulating HIF-1α dynamics (Figure 5C). Knockout of either miR-155 or PHD2 is mimicked by setting the corresponding HIF-1α-dependent induction rates to zero. At 1% *O*_2_, [HIF-1α_tot_] still shows adaptive dynamics with a higher peak in the case of PHD2 knockout. With miR-155 knockout, [HIF-1α_tot_] settles at a plateau instead of showing adaptation, consistent with the experimental results (Bruning et al., 2011). In the case of both miR-155 and PHD2 knockout, [HIF-1α_tot_] stays at higher levels persistently compared to the case of miR-155 knockout alone. Therefore, our results suggest that miR-155 is required for the adaptive dynamics of HIF-1α while PHD2 mainly contributes to the suppression of HIF-1α accumulation.

It is intriguing to investigate the effect of miR-155 abundance on HIF-1α dynamics in severe hypoxia. We show the above effect of miR-155 by plotting the curves of [HIF-1α_tot_] dynamics for various *k*_smiR1551_ (the induction rate of miR-155 by unhydroxylated HIF-1α) at 0.1% O_2_ (Figure 5D). As shown previously, HIF-1α_tot_ stays at high levels in the default case. For smaller *k*_smiR1551_, [HIF-1α_tot_] rises to higher levels; for larger *k*_smiR1551_, [HIF-1α_tot_] stays at lower levels; for further increased *k*_smiR1551_, [HIF-1α_tot_] cannot keep at high levels and drops to basal levels, exhibiting adaptive dynamics (Figure 5D). Together, miR-155 modulates HIF-1α dynamic modes markedly under severe hypoxia, and its overexpression can transform HIF-1α dynamics to adaptive mode.

### 3.4. Crosstalk of lincRNA-p21 and miR-155 in Shaping HIF-1α Dynamics

**Figure 6:**
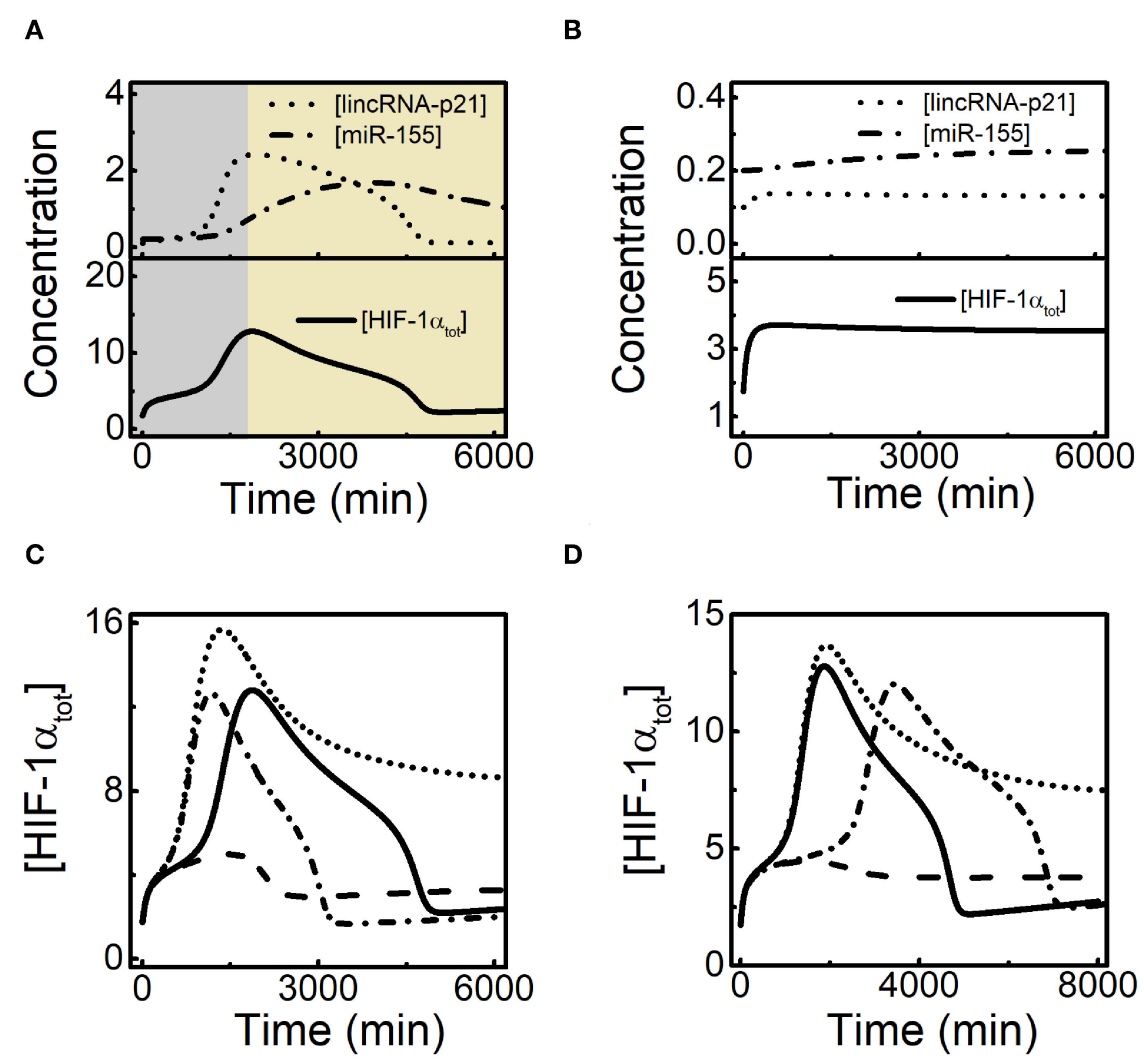
Interplay between lincRNA-p21 and miR-155 in the adaptative dynamics of HIF-1α. **(A)** Time courses of [lincRNA-p21] (dotted), [miR-155] (dash-dotted), and [HIF-1α_tot_] (solid) at 1% *O*_2_ in the default case. **(B)** Time courses of [lincRNA-p21] (dotted), [miR-155] (dash-dotted), and [HIF-1α_tot_] (solid) at 1% *O*_2_ with decreased lincRNA-p21 induction rate (*k*_sLAp21a_ = 0.02). **(C)** Time courses of [HIF-1α_tot_] at 1% *O*_2_ with the standard parameter setting (solid, *k*_sLAp21a_ = 0.05 and *k*_smiR1551a_ = 0.0012), increased lincRNA-p21 induction rate alone (dotted, *k*_sLAp21a_ = 0.07, *k*_smiR1551a_ = 0.0012), increased miR-155 expression alone (dashed, *k*_sLAp21a_ = 0.05 and *k*_smiR1551a_ = 0.005), and both increased lincRNA-p21 and miR-155 expression (dash-dotted, *k*_sLAp21a_ = 0.07 and *k*_smiR1551a_ = 0.005). **(D)** Time courses of [HIF-1α_tot_] at 1% *O*_2_ for the default case (solid, *k*_sLAp21a_ = 0.05 and *k*_smiR1551a_ = 0.0012), decreased miR-155 expression alone (dotted, *k*_sLAp21a_ = 0.05 and *k*_smiR1551a_ = 0.0007), decreased lincRNA-p21 expression alone (dashed, *k*_sLAp21a_ = 0.043 and *k*_smiR1551a_ = 0.0012), and both decreased lincRNA-p21 and miR-155 expression (dash-dotted, *k*_sLAp21a_ = 0.043 and *k*_smiR1551a_ = 0.0007).

Since both lincRNA-p21 and miR-155 are involved in HIF-1α-centered feedback loops, there may exist crosstalk between them in modulating HIF-1α dynamics. The dynamic curves of [lincRNA-p21], [miR-155], and [HIF-1α_tot_] are plotted together to show their temporal evolution during the hypoxic response (Figure 6A). LincRNA-p21 and miR-155 are separately induced by HIF-1α so that lincRNA-p21 stimulates the rising of HIF-1α in the early phase while miR-155 renders the recovery of HIF-1α in the late phase. With decreased *k*_sLAp21a_, lincRNA-p21 settles at low state instead of showing adaptive dynamics (Figure 6B). As a result, [HIF-1α_tot_] stays at rather low levels and miR-155 is not induced markedly without enough HIF-1α. Our results reveal that sufficient induction of lincRNA-p21 by HIF-1α is required for subsequent induction of miR-155 and recovery of HIF-1α.

We further explore how lincRNA-p21 and miR-155 interplay to modulate the adaptive dynamics of HIF-1α. We compare the dynamics of [HIF-1α_tot_] by increasing the induction rates of lincRNA-p21 and (or) miR-155 (Figure 6C). For increased *k*_sLAp21a_, lincRNA-p21 counteracts the recovery of HIF-1α by miR-155, and [HIF-1α_tot_] maintains at moderate levels in the late phase. When *k*_smiR1551a_ is also enlarged, [HIF-1α_tot_] restores prefect adaptation to hypoxia. Likewise, with increasing *k*_smiR1551a_ alone, overexpressed miR-155 suppresses the stabilization of HIF-1α by lincRNA-p21 and [HIF-1α_tot_] only shows a small pulse. We also show the effects of decreasing *k*_sLAp21a_ and/or *k*_smiR1551a_ on HIF-1α dynamics (Figure 6D). When *k*_smiR1551a_ alone is decreased, [HIF-1α_tot_] stays at moderate levels; while decreasing *k*_sLAp21a_ alone, it only rises to low levels; adaptive dynamics of [HIF-1α_tot_] reappears in the case of decreasing both *k*_sLAp21a_ and *k*_smiR1551a_. Together, lincRNA-p21 and miR-155 cooperate in shaping HIF-1α dynamics and their balance is critical for the perfect adaptation of HIF-1α to hypoxia.

## 4. Conclusion and Discussion

We have built a network model to probe how HIF-1α-targeted lincRNA-p21 and miR-155 coordinate to regulate the adaption of HIF-1α to hypoxia. We found that lincRNA-p21 and miR-155 are sequentially induced during hypoxia. LincRNA-p21 promotes the rising of HIF-1α by stabilizing it in the early phase, while miR-155 promotes the recovery of HIF-1α in the late phase by facilitating the degradation of its mRNA. Moreover, there exists a delicate balance between lincRNA-p21 and miR-155 in shaping HIF-1α dynamics: variation in the adaptive dynamics of HIF-1α due to changes in the expression of either ncRNA can be counteracted by changing the expression of the other.

It has been shown that both HIF-1α and lincRNA-p21 exhibit adaptive dynamics in response to hypoxia (Yang et al., 2014). How they are down-regulated in the late phase of the response is not well-elucidated. We proposed that HIF-1α-induced miR-155 may contribute to the recovery of HIF-1α and lincRNA-p21 to low levels. In addition, we also reveal that lincRNA-p21 promotes the rising of HIF-1α in the early phase via stabilizing it. Furthermore, we indicate that the stabilization of partially activated proline-unhydroxylated HIF-1α by lincRNA-p21 plays a dominant role in promoting HIF-1α accumulation. Therefore, coordination of HIF-1α-centered positive and negative feedback loops ensures the adaptive adaption of HIF-1α to hypoxia.

We assumed that lincRNA-p21 can repress the degradation of proline-unhydroxylated HIF-1α. The assumption is supported by the experimental evidence that HIF-1α induces lincRNA-p21 to stabilize the proline-unhydroxylated HIF-1α by disrupting the interaction with VHL and enhances its own transcriptional activity remarkably (Yang et al., 2014). It has been reported that two forms of proline-unhydroxylated HIF-1α, i.e., unhydroxylated HIF-1α and HIF-1α with asparaginyl-hydroxylation alone, can transactivate the target genes (Dayan et al., 2006). Moreover, given proline-hydroxylated HIF-1α loses its transcriptional activity and prolyl-hydroxylation is considered to be irreversible (Schofield and Ratcliffe, 2004; Chan et al., 2005), lincRNA-p21-dependent accumulation of proline-hydroxylated HIF-1α has no contribution to induction of target genes. Therefore, it is plausible to assume that lincRNA-p21 enhances the transcriptional activity of HIF-1α by stabilizing the proline-unhydroxylated forms. It has been reported that lincRNA-p21 promotes HIF-1α-dependent glycolysis via inducing several target genes (Yang et al., 2014). Nevertheless, the interaction between HIF-1α and VHL is rather weak, and the detailed mechanism underlying the upregulation of HIF-1α activity by lincRNA p21 is to be further investigated.

Our work reveals that miR-155 and PHD2 play non-redundant roles in promoting HIF-1α recovery in hypoxia. Our results show that PHD2 mainly modulates the peak level of HIF-1α in the adaptive dynamics, consistent with the modeling results reported by Fábián et al. (2016). On the other hand, it has been suggested that PHD2 mainly regulates HIF-1α in the early phase in contrast to miR-155 (Bruning et al., 2011). We proposed that HIF-1α-miR-155 and HIF-1α-PHD2 negative feedback loops play the main and auxiliary role respectively in adaptive response of HIF-1α to hypoxia. Nevertheless, PHD2 may play a significant role in the adaptive dynamics of HIF-1α in some other cell lines (Ginouvés et al., 2008; Bagnall et al., 2014). Therefore, the roles of PHD2 and miR-155 may be context-dependent or cell-type specific.

LincRNA-p21 and miR-155 regulate HIF-1α positively and negatively in the separate phases to ensure the adaptive dynamics. They should be induced and predominate in the early and late phase of the response, respectively (Bruning et al., 2011; Yang et al., 2014). There may exist a delicate balance between lincRNA-p21 and miR-155 in shaping HIF-1α dynamics. For increased lincRNA-p21 expression, dominance of miR-155 is weakened in the late phase so that HIF-1α cannot return to low levels entirely. Increased miR-155 expression also impairs the balance between it and lincRNA-p21, suppressing HIF-1α accumulation. Therefore, when the expression of either one is changed, the other needs to be varied in the same direction to guarantee the perfect adaptation of HIF-1α to hypoxia. In addition, under severe hypoxia, enhancing lincRNA-p21 induction impairs the balance in the regulation of HIF-1α, so HIF-1α settles down at relatively high levels. We propose that repressing lincRNA-p21 or increasing miR-155 expression may facilitate the adaptation of HIF-1α under serious hypoxia.

It has been indicated that tight control of transient HIF-1α dynamics is crucial for cell survival (Bagnall et al., 2014). LincRNA-p21 and miR-155 have the potential to modulate cellular outcome in the hypoxic response since they can modulate the dynamic modes of HIF-1α. On one hand, knocking down lincRNA-p21 may be a rational strategy for repressing tumorigenesis as lincRNA-p21 can promote HIF-1α accumulation and facilitate the adaptation of tumors to hypoxia (Yang et al., 2014; Koyasu et al., 2018). Indeed, lincRNA-p21 knockdown induces G2/M phase arrest and promotes apoptosis to enhance the radiosensitivity of SMMC7721 and U251MG cells in hypoxia (Shen et al., 2017). Moreover, it has been reported that lincRNA-p21 knockout abrogates the migration and survival of mesenchymal stem cells by hypoxia preconditioning (Meng et al., 2018). On the other hand, miR-155 mediates proliferation suppression of non-small cell lung cancer cells in radiotherapy via decreasing the expression of HIF-1α (Zhu et al., 2019). miR-155 deficiency results in upregulated HIF-1α expression, promoting the growth of solid tumors (Wang et al., 2015). In addition, a recent study showed that targeting the temporal dynamics of HIF-1α-induced tumor-secreted microenvironmental factors can halt tumor migration (Singh et al., 2019). Lehmann et al. revealed that hypoxia can induce a HIF-1α-dependent transition from collective-to-amoeboid dissemination in epithelial cancer cells (Lehmann et al., 2017). Kang et al. proposed that HIF-1α and several microRNAs including miR-34, miR-145, and miR-200, may play critical roles in epithelial to mesenchymal transition and cancer metastasis (Kang et al., 2019). Thus, we predict that modulating HIF-1α dynamics or activity by changing lincRNA-p21 or miR-155 expression may affect cancer migration and dissemination remarkably. Together, more attention should be paid to the treatment strategy of cancer by targeting lincRNA-p21 and miR-155 in the future.

## Data Availability Statement

All datasets generated for this study are included in the article/Supplementary Material.

## Author Contributions

X-PZ conceived and designed the research. C-YS carried out computer implementation and wrote the original manuscript. C-YS and X-PZ analyzed and interpreted the simulation results. X-PZ and FL supervised the project. C-YS, X-PZ, FL, and WW revised and contributed to the writing of final manuscript. All authors contributed to the article and approved the submitted version.

## Conflict of Interest

The authors declare that the research was conducted in the absence of any commercial or financial relationships that could be construed as a potential conflict of interest.
