# Supplementary material for: Orchestration of lincRNA-p21 and miR-155 in Modulating the Adaptive Dynamics of HIF-1α
Source: Front Genet. 2020 Aug 18;11:871. doi: 10.3389/fgene.2020.00871 (PMC7461903; doi:10.3389/fgene.2020.00871)
Supplement: Supplementary file 1 [file Presentation_1.pdf]

SUPPORTING MATERIAL

Non-coding lincRNA-p21 and microRNA-155 Mediate the cellular adaptation to hypoxia

Here, we present the ordinary differential equations for the model and standard parameter values and the initial values of variables .

Appendix: Ordinary Differential Equations for the Model

$$\frac{d[\text{HIF-1αm}]}{dt} = k_{\text{shifm}} - k_{\text{dhifm}}[\text{HIF-1αm}] \tag{1}$$

$$k_{\text{dhifm}} = k_{\text{dhifm0}} + k_{\text{dhifm1}}\frac{[\text{miR-155}]}{[\text{miR-155}] + j_{\text{55hifmd}}} \tag{2}$$

$$\frac{d[\text{FIH-1}_{\text{ac}}]}{dt} = k_{\text{acfih}}\frac{O_2}{O_2 + j_{\text{o2fih}}}[\text{FIH-1}] - k_{\text{defih}}[\text{FIH-1}_{\text{ac}}] \tag{3}$$

$$[\text{FIH-1}] = [\text{FIH-1}_{\text{tot}}] - [\text{FIH-1}_{\text{ac}}] \tag{4}$$

$$\frac{d[\text{HIF-1α}]}{dt} = k_{\text{thif}}[\text{HIF-1αm}] - k_{\text{dhif}}\frac{j_{\text{dhif}}}{j_{\text{dhif}} + [\text{lincRNA-p21}]}[\text{HIF-1α}]$$
$$- k_{\text{dhiffih}}\frac{j_{\text{dhiffih}}}{j_{\text{dhiffih}} + [\text{FIH-1}_{\text{ac}}]}[\text{HIF-1α}] - k_{\text{aoh}}[\text{FIH-1}_{\text{ac}}]\frac{[\text{HIF-1α}]}{[\text{HIF-1α}] + j_{\text{hiffih}}} \tag{5}$$

$$\frac{d[\text{HIF-1α-aOH}]}{dt} = k_{\text{aoh}}[\text{FIH-1}_{\text{ac}}]\frac{[\text{HIF-1α}]}{[\text{HIF-1α}] + j_{\text{hiffih}}} - k_{\text{poh}}[\text{PHD2}_{\text{ac}}]\frac{[\text{HIF-1α-aOH}]}{[\text{HIF-1α-aOH}] + j_{\text{hifphd}}}$$
$$-k_{\text{dhif}}\frac{j_{\text{dhif}}}{j_{\text{dhif}} + [\text{lincRNA-p21}]}[\text{HIF-1α-aOH}] \tag{6}$$

$$\frac{d[\text{HIF-1α-aOHpOH}]}{dt} = k_{\text{poh}}[\text{PHD2}_{\text{ac}}]\frac{[\text{HIF-1α-aOH}]}{[\text{HIF-1α-aOH}] + j_{\text{hifphd}}}$$
$$-k_{\text{dhifpoh}}\frac{j_{\text{dhifpoh}}}{j_{\text{dhifpoh}} + [\text{lincRNA-p21}]}[\text{HIF-1α-aOHpOH}] \tag{7}$$

$$\frac{d[\text{PHD2}_{\text{tot}}]}{dt} = k_{\text{sphd0}} + k_{\text{sphd1}}\frac{[\text{HIF-1α}]^n}{[\text{HIF-1α}]^n + j_{\text{sphd1}}^n} + k_{\text{sphd1a}}\frac{[\text{HIF-1α-aOH}]^n}{[\text{HIF-1α-aOH}]^n + j_{\text{sphd1a}}^n}$$
$$-k_{\text{dphd}}[\text{PHD2}_{\text{tot}}] \tag{8}$$

$$\frac{d[\mathrm{PHD2_{ac}}]}{dt} = k_{\mathrm{acphd}} \frac{O_2}{O_2 + j_{\mathrm{o2phd}}} [\mathrm{PHD2}] - k_{\mathrm{dephd}} [\mathrm{PHD2_{ac}}] \tag{9}$$

$$[\mathrm{PHD2}] = [\mathrm{PHD2_{tot}}] - [\mathrm{PHD2_{ac}}] \tag{10}$$

$$\frac{d[\text{miR-155}]}{dt} = k_{\mathrm{smiR1550}} + k_{\mathrm{smiR1551}} \frac{[\text{HIF-1}\alpha]^n}{[\text{HIF-1}\alpha]^n + j_{\mathrm{smiR1551}}^n}$$

$$+k_{\mathrm{smiR1551a}} \frac{[\text{HIF-1}\alpha\text{-aOH}]^n}{[\text{HIF-1}\alpha\text{-aOH}]^n + j_{\mathrm{smiR1551a}}^n} - k_{\mathrm{dmiR155}} [\text{miR-155}] \tag{11}$$

$$\frac{d[\text{lincRNA-p21}]}{dt} = k_{\mathrm{sLAp210}} + k_{\mathrm{sLAp21}} \frac{[\text{HIF-1}\alpha]^n}{[\text{HIF-1}\alpha]^n + j_{\mathrm{sLAp21}}^n}$$

$$+k_{\mathrm{sLAp21a}} \frac{[\text{HIF-1}\alpha\text{-aOH}]^n}{[\text{HIF-1}\alpha\text{-aOH}]^n + j_{\mathrm{sLAp21a}}^n} - k_{\mathrm{dLAp21}} [\text{lincRNA-p21}] \tag{12}$$

Table S1: Variables and their initial values

Variable	initial values	Variable	initial values	Variable	initial values
[HIF-1αm]	1.5	[HIF-1α-aOH]	1.04	$[\mathrm{PHD2_{ac}}]$	2.3
$[\text{FIH-1}_{\mathrm{ac}}]$	6.92	[HIF-1α-aOHpOH]	0.14	[miR-155]	0.2
[HIF-1α]	0.56	$[\mathrm{PHD2_{tot}}]$	5	[lincRNA-p21]	0.1

Table S2: Standard Parameter Values

Parameter	Description	Value	Reference
k_{shifm}	Induction rate of *HIF-1α* mRNA	0.0053	estimated
k_{dhifm0}	Basal degradation rate of *HIF-1α* mRNA	0.003	[1]
k_{dhifm1}	miR-155-dependent degradation rate of *HIF-1α* mRNA	0.006	estimated
j_{55hifmd}	Michaelis constant of miR-155-dependent *HIF-1α* mRNA degradation	2	estimated
$[\text{FIH-1}_{\mathrm{tot}}]$	Total concentration of FIH-1	10	estimated
k_{acfih}	O_2-dependent activation rate of FIH-1	3	estimated
j_{o2fih}	Michaelis constant of O_2-dependent FIH-1 activation	7	[2,3]
k_{defih}	Deactivation rate of FIH-1	1	estimated
k_{thif}	Translation rate of *HIF-1α* mRNA	0.04	estimated
k_{dhif}	Primary degradation rate of HIF-1α or HIF-1α-aOH	0.02	[4]
j_{dhif}	Michaelis constant of repressing HIF-1α or HIF-1α-aOH degradation by lincRNA-p21	0.3	estimated
k_{poh}	PHD2-induced hydroxylation rate of HIF-1α-aOH at proline residue	0.06	estimated
j_{hifphd}	Michaelis constant for HIF-1α-aOH as a substrate of PHD2	3	estimated
k_{aoh}	FIH-1-induced hydroxylation rate of HIF-1α at asparagine residue	0.06	estimated
j_{hiffih}	Michaelis constant for HIF-1α as a substrate of FIH-1	4	estimated
k_{dhiffih}	FIH-1-related degradation rate of HIF-1α	0.0032	estimated
j_{dhiffih}	Michaelis constant of FIH-1-related HIF-1α degradation	0.1	estimated

k_{dhifpoh}	Primary degradation rate of HIF-1α-aOHpOH	0.34	[4]
j_{dhifpoh}	Michaelis constant of repressing HIF-1α-aOHpOH degradation by lincRNA-p21	0.3	estimated
n	Hill coefficient of transactivating PHD2, miR-155 and lincRNA-p21 by HIF-1α or HIF-1α-aOH	4	estimated
k_{sphd0}	Basal induction rate of PHD2	0.03	estimated
k_{sphd1}	HIF-1α-inducible production rate of PHD2	0.03	estimated
j_{sphd1}	Michaelis constant of HIF-1α-dependent PHD2 production	6	estimated
k_{sphd1a}	HIF-1α-aOH-inducible production rate of PHD2	0.02	estimated
j_{sphd1a}	Michaelis constant of HIF-1α-aOH-dependent PHD2 production	10	estimated
k_{dphd}	Degradation rate of PHD2	0.006	estimated
k_{acphd}	O_2-dependent activation rate of PHD2	1.8	estimated
j_{o2phd}	Michaelis constant of O_2-dependent PHD2 activation	23	[2,3]
k_{dephd}	Deactivation rate of PHD2	1	estimated
k_{smiR1550}	Basal induction rate of miR-155	6.4E-5	estimated
k_{smiR1551}	HIF-1α-inducible production rate of miR-155	0.0024	estimated
j_{smiR1551}	Michaelis constant of HIF-1α-dependent miR-155 production	6.5	estimated
$k_{\mathrm{smiR1551a}}$	HIF-1α-aOH-inducible production rate of miR-155	0.0012	estimated
$j_{\mathrm{smiR1551a}}$	Michaelis constant of HIF-1α-aOH-dependent miR-155 production	6.5	estimated
k_{dmiR155}	Degradation rate of miR-155	0.00032	[5]
k_{sLAp210}	Basal induction rate of lincRNA-p21	0.002	estimated
k_{sLAp21}	HIF-1α-inducible production rate of lincRNA-p21	0.08	estimated
j_{sLAp21}	Michaelis constant of HIF-1α-dependent lincRNA-p21 production	5.7	estimated
k_{sLAp21a}	HIF-1α-aOH-inducible production rate of lincRNA-p21	0.05	estimated
j_{sLAp21a}	Michaelis constant of HIF-1α-aOH-dependent lincRNA-p21 production	5.8	estimated
k_{dLAp21}	Degradation rate of lincRNA-p21	0.02	estimated

Note: it is the relative rather than absolute value of O_2 level that makes sense, so for convenience, the oxygen level at normoxia is denoted as 21 and j_{o2phd} and j_{o2fih} are determined according to the relation between the K_m of PHD2 and FIH-1 activation for O_2 [2,3].

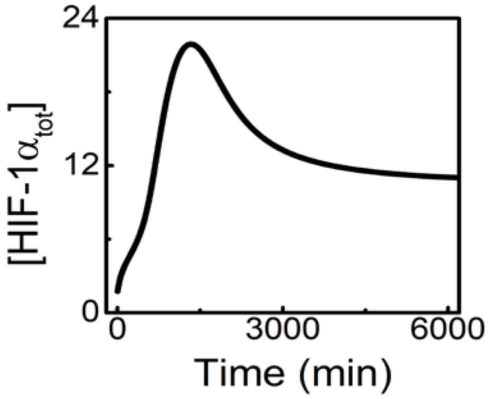

Figure S1: Time courses of [HIF-1α_{tot}] for 0.3% O_2

References

[1] Bett, J. S., Ibrahim, A. F., et al. 2013. The P-body component USP52/PAN2 is a novel regulator of HIF1A mRNA stability. Biochem J 451(2): 185-194

[2] Koivunen, P., Hirsia, M., et al. 2004. Catalytic properties of the asparaginyl hydroxylase (FIH) in the oxygen sensing pathway are distinct from those of its prolyl 4-hydroxylases. J Biol Chem 279(11): 9899-9904.

[3] Qutub, A. A. and A. S. Popel (2006). "A computational model of intracellular oxygen sensing by hypoxia-inducible factor HIF1 alpha." J Cell Sci 119(Pt 16): 3467-3480.

[4] Huang, L. E., Gu, J., Schau, M. and Bunn, H. F. 1998. Regulation of hypoxia inducible factor 1α is mediated by an O2-dependent degradation domain via the ubiquitin-proteasome pathway. Proc. Natl. Acad. Sci. USA 95, 7987-7992.

[5] Gantier, M. P. 2010. New Perspectives in MicroRNA Regulation of Innate Immunity. Journal of Interferon & Cytokine Research 30: 283-289
